# The Association Between Financial Toxicity and Treatment Regret in Men With Localized Prostate Cancer

**DOI:** 10.1093/jncics/pkac071

**Published:** 2022-10-18

**Authors:** Daniel D Joyce, Christopher J D Wallis, Li-Ching Huang, Karen E Hoffman, Zhiguo Zhao, Tatsuki Koyama, Michael Goodman, Ann S Hamilton, Xiao-Cheng Wu, Lisa E Paddock, Antoinette Stroup, Matthew R Cooperberg, Mia Hashibe, Brock B O’Neil, Sherrie H Kaplan, Sheldon Greenfield, David F Penson, Daniel A Barocas

**Affiliations:** Department of Urology, Mayo Clinic, Rochester, MN, USA; Department of Surgery, Division of Urology, University of Toronto, Toronto, ON, Canada; Department of Biostatistics, Vanderbilt University Medical Center, Nashville, TN, USA; Department of Radiation Oncology, The University of Texas, MD Anderson Cancer Center, Houston, TX, USA; Department of Biostatistics, Vanderbilt University Medical Center, Nashville, TN, USA; Department of Biostatistics, Vanderbilt University Medical Center, Nashville, TN, USA; Department of Epidemiology, Emory University Rollins School of Public Health, Atlanta, GA, USA; Department of Population and Public Health Sciences, Keck School of Medicine at the University of Southern California, Los Angeles, CA, USA; Department of Epidemiology, Louisiana State University New Orleans School of Public Health, New Orleans, LA, USA; Department of Epidemiology, Cancer Institute of New Jersey, Rutgers Health, New Brunswick, NJ, USA; Department of Epidemiology, Cancer Institute of New Jersey, Rutgers Health, New Brunswick, NJ, USA; Department of Urology, University of California, San Francisco, CA, USA; Department of Family and Preventative Medicine, University of Utah School of Medicine, Salt Lake City, UT, USA; Department of Urology, University of Utah Health, Salt Lake City, UT, USA; Department of Medicine, University of California Irvine, Irvine, CA, USA; Department of Medicine, University of California Irvine, Irvine, CA, USA; Department of Urology, Vanderbilt University Medical Center, Nashville, TN, USA; Geriatric Research Education and Clinical Center, Veterans Affairs Tennessee Valley Healthcare System, Nashville, TN, USA; Department of Urology, Vanderbilt University Medical Center, Nashville, TN, USA

## Abstract

**Background:**

Financial toxicity is emerging as an important patient-centered outcome and is understudied in prostate cancer patients. We sought to understand the association between financial burden and treatment regret in men with localized prostate cancer to better evaluate the role of financial discussions in patient counseling.

**Methods:**

Utilizing the Comparative Effectiveness Analysis of Surgery and Radiation dataset, we identified all men accrued between 2011 and 2012 who underwent surgery, radiation, or active surveillance for localized prostate cancer. Financial burden and treatment regret were assessed at 3- and 5-year follow-up. The association between financial burden and regret was assessed using multivariable longitudinal logistic regression controlling for demographic and disease characteristics, treatment, functional outcomes, and patient expectations.

**Results:**

Of the 2924 eligible patients, regret and financial burden assessments for 3- and/or 5-year follow-up were available for 81% (n = 2359). After adjustment for relevant covariates, financial burden from “finances in general” was associated with treatment regret at 3 years (odds ratio [OR] = 2.47, 95% confidence interval [CI] = 1.33 to 4.57; *P* = .004); however, this association was no longer statistically significant at 5-year follow-up (OR = 1.19, 95% CI = 0.56 to 2.54; *P* = .7).

**Conclusions:**

In this population-based sample of men with localized prostate cancer, we observed associations between financial burden and treatment regret. Our findings suggest indirect treatment costs, especially during the first 3 years after diagnosis, may impact patients more profoundly than direct costs and are important for inclusion in shared decision making.

Management of localized prostate cancer presents challenges for clinicians given the multitude of treatment options with often comparable oncologic but varying functional outcomes. Management choice ultimately results from a shared decision-making process between the provider and patient. This process involves assessing patient values and severity of disease and providing accurate expectations for treatment functional side effects and impact on quality of life. Failure of treatment outcomes to meet expectations may result in treatment regret, which can negatively impact a patient’s mental health and health-related quality of life (1,2).

Financial toxicity, a term used to describe the negative effects of treatment costs on patients, is an increasingly recognized, important patient-centered outcome. Just as urinary incontinence can negatively impact quality of life following prostatectomy, filing for bankruptcy, involuntary early retirement, depletion of savings, and foregoing basic needs to pay for cancer treatment can similarly negatively impact a patient’s quality of life (3,4). Despite evidence of the relationship between financial toxicity and symptom burden, health-related quality of life, and even mortality, its inclusion in treatment decision-making processes is rare (5).

Little is known regarding how financial toxicity may or may not influence treatment regret. We sought to investigate the associations between 2 financial toxicity components—direct and indirect costs—and treatment regret among men with localized prostate cancer. We hypothesized that patients who expressed higher levels of financial burden following initial management of their disease would be more likely to report treatment regret. Through addressing this gap in the current literature, we hope to highlight the importance of inclusion of financial discussions in shared decision-making processes and guide future research aimed at alleviating financial toxicity in this population.

## Methods

### Patient Population

The study sample was obtained from the prospective population-based cohort Comparative Effectiveness Analyses of Surgery and Radiation (CEASAR) study, which accrued between 2011 and 2012 and included men undergoing management of localized prostate cancer at 5 Surveillance, Epidemiology, and End Results registries and the Cancer of Prostate Strategic Urologic Research Endeavour registry (6). Institutional review board approvals were obtained from the coordinating site (Vanderbilt University Medical Center) as well as all participating sites.

The CEASAR study included men aged 80 years and younger diagnosed with localized prostate cancer (cT1-T2N0M0) within 6 months of enrollment and prostate-specific antigen of less than 50 ng/mL. For our analysis, we included all men who completed the financial burden and treatment regret survey questions at either 3- or 5-year follow-up (from the time of baseline survey). We excluded study participants from the Cancer of Prostate Strategic Urologic Research Endeavour registry and whose primary treatment was either ablation therapy or hormone-directed monotherapy to limit our analysis to the most common and generalizable management strategies.

Mailed surveys were completed at baseline, 6 months, 1 year, 3 years, and 5 years after enrollment (Supplementary Figure 1, available online). Multiple methods were employed to encourage response including telephone follow-up, postcard reminders, re-mailing survey packets, and when needed, a phone interview administered by a trained research staff member. All patient-reported data were supplemented by medical chart and cancer registry abstraction at 1 year following study enrollment. Inter-rater reliability and validity of the medical chart abstraction process have been previously described (6).

### Measurement and Definition of Financial Burden

Patient responses to 4 items within the CEASAR questionnaire were used to assess the primary exposure of interest: financial burden from direct and indirect costs (Supplementary Table 1, available online) (7). These questions were adapted by the study methodologist and psychometrician (SHK) from a disease burden scale originally developed for the assessment of medical outcomes among differing medical practices and previously used in diabetes research (8-10). Responses to each item were converted to a binary variable with financial burden defined as a response of “very large” or “large.” Three of these items were designed to assess the financial burden associated with specific direct costs (cost of treatment, cost of health care for prostate cancer, and cost of health insurance), and the fourth item was designed to measure overall burden (finances in general), which includes these direct costs as well as any additional indirect costs.

### Measurement and Definition of Treatment Regret

Our primary outcome of interest was patient-reported treatment regret. This was assessed at 3- and 5-year follow-up using prostate cancer–specific scale described by Clark et al. (11) Scaled scoring and sum transformation were performed according to Clark and colleagues’ methodology with final scores ranging from 0 to 100. Patients with scores of 40 or higher were considered to have clinically significant treatment regret.

### Potential Confounders and Effect Modifiers

Additional patient, disease, and treatment characteristics that may be associated with financial burden and treatment regret were included in our analyses. Patient-reported variables included age at diagnosis, race and ethnicity, income, employment status, education, and marital status. Type of health insurance was similarly obtained from patient surveys and reported as Medicare, which included those patients with dual private and Medicare coverage, private or health maintenance organization, Veteran Affairs or military, Medicaid, other insurance, and no insurance.

Disease characteristics such as D’Amico risk group, clinical tumor stage, prostate-specific antigen at diagnosis, biopsy Gleason score, and treatment type were obtained from medical record or cancer registry data abstraction at 1 year following enrollment. Comorbidity was assessed using the total illness burden index for prostate cancer (12).

Functional outcomes of prostate cancer management were included, given the potential mediating effect on treatment regret, and were assessed at each follow-up time point using the 26-item Expanded Prostate Index Composite (EPIC-26) questionnaire. Scores were calculated for each of the 5 EPIC domains—hormonal, sexual, urinary incontinence, urinary irritative, and bowel—and ranged from 0 to 100 with higher scores signifying better function. Health-related quality of life was similarly assessed at each time point using the validated Medical Outcomes Study 36-item Short Form Survey (SF-36) (13,14).

Potential associations between regret and patient expectations were accounted for through assessment of differences between experienced and expected treatment efficacy and toxicity outcomes using a 5-point Likert scale as described elsewhere (15).

### Statistical Analysis

Patients’ demographic and clinical disease characteristics were compared between those with and without overall financial burden at 3- and 5-year surveys. Continuous variables were summarized using medians (quartiles) and compared with Wilcoxon rank tests; categorical variables were summarized using frequencies (percentages) and compared with χ^2^ tests.

Financial burden was estimated with the following 4 individual items: 1 for overall financial burden (finances in general) and 3 for burden from direct costs (treatment costs, health care for prostate cancer, and health insurance). To evaluate the associations between these 4 individual items and treatment regret, multivariable longitudinal logistic regression models were used. Because these 4 financial burden items are highly correlated, we analyzed these associations in separate models to avoid multicollinearity. Our primary analysis was to explore the association between finances in general and treatment regret. Associations between the remaining financial burden items and treatment regret were treated as secondary analyses and should be interpreted as exploratory. To account for the potential serial correlation between the 2 records collected at 3 and 5 years from each patient, generalized estimating equations were used with the Huber-White method to estimate robust covariance matrix. The results were reported as adjusted odds ratios (aORs) with 95% confidence intervals (CIs). The 4 models accounted for each of the potential confounders described above as well as survey time (3 years, 5 years). For the 4 individual financial burden items, we included the interaction terms with the survey time (3 or 5 years) to allow the individual item-regret association to vary over time. For the functional outcomes (5 domain scores), the changes from the baseline scores were analyzed in all models. Age was modeled with restricted cubic splines with 3 knots to allow for nonlinear association with the outcome.

Missing values in the regression variables were imputed using the multiple imputation using chained equations procedure (16,17). No outcome variables were imputed. Two-sided *P* values less than .05 were consider statistically significant. All analyses were conducted using R version 4.1.

## Results

**Figure 1.**
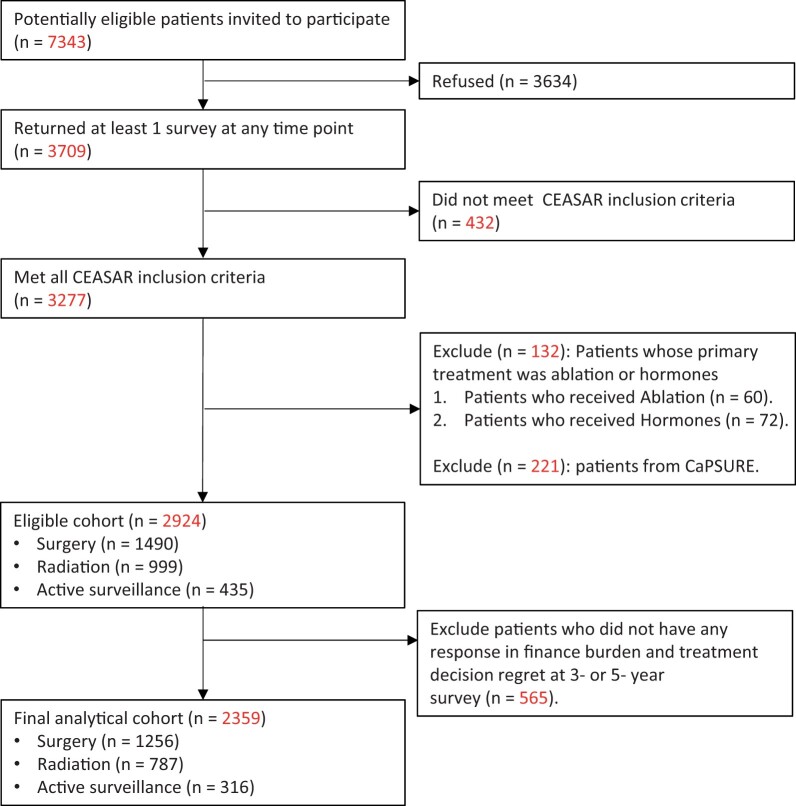
Study population flow diagram. CaPSURE = Cancer of Prostate Strategic Urologic Research Endeavour; CEASAR = Comparative Effectiveness Analyses of Surgery and Radiation.

Of the 2924 eligible patients, 2359 (81%) completed financial burden and treatment regret surveys at either 3- or 5-year follow-up (Figure 1). Financial burden surveys were completed by 2252 study patients (95%) at 3-year survey and 2051 (87%) at 5-year survey. Treatment among these men included surgery (n = 1256, 53%), radiation (n = 787, 33%), and active surveillance (n = 316, 13%). Overall, 138 of 2359 unique patients experienced financial burden at either 3- or 5-year follow-up. At 3- and 5-year survey, 97 (4.3%) and 73 (3.6%) patients expressed large or very large overall financial burden, respectively. These men were more likely to be younger, non-White, unemployed, less educated, and not married and have lower annual household income, higher total illness burden index scores, and higher risk disease compared with those without financial burden (Table 1). Additionally, men with financial burden were more likely to describe treatment effectiveness as “a lot worse” compared with expectations (8% vs 2% at 3 years and 7% vs 1% at 5 years; both *P* < .001) and side effects of treatment as “a lot worse” compared with expectations (21% vs 10% at 3 years; *P* < .001; and 19% vs 10% at 5 years; *P* = .02).

**Table 1.** Comparative patient and disease characteristics between those expressing “very large” or “large” financial burden from finances in general and those expressing “neutral,” “small,” “very small,” or “no” financial burden at 3 and 5 years

	Financial burden at 3 years				Financial burden at 5 years			
Characteristics	Very large/large burden	Neutral/small/very small/no burden	Combined	*P*	Very large/large burden	Neutral/small/very small/no burden	Combined	*P*
	(n = 97)	(n = 2155)	(n = 2252)		(n = 73)	(n = 1978)	(n = 2051)	
Age at diagnosis, median (IQR) y	60 (55, 66)	64 (59, 70)	64 (59, 70)	<.001	62 (55, 66)	64 (59, 69)	64 (59, 70)	.002
Race and ethnicity, No. (%)								
Asian	5 (5)	62 (3)	67 (3)	<.001	3 (4)	61 (3)	64 (3)	<.001
Black	25 (26)	272 (13)	297 (13)		19 (26)	233 (12)	252 (12)	
Hispanic	14 (14)	137 (6)	151 (7)		17 (23)	123 (6)	140 (7)	
White	52 (54)	1654 (77)	1706 (76)		33 (45)	1531 (77)	1564 (76)	
Other^a^	1 (1)	29 (1)	30 (1)		1 (1)	29 (1)	30 (1)	
Income, No. (%)								
<$30 000	54 (62)	328 (17)	382 (19)	<.001	39 (59)	301 (17)	340 (18)	<.001
$30 001-$50 000	15 (17)	380 (19)	395 (19)		12 (18)	338 (19)	350 (19)	
$50 001-$100 000	11 (13)	634 (33)	645 (32)		9 (14)	586 (33)	595 (32)	
>$100 000	7 (8)	607 (31)	614 (30)		6 (9)	577 (32)	583 (31)	
Employment, No. (%)								
Full time	33 (35)	929 (43)	962 (43)	<.001	24 (34)	875 (45)	899 (44)	<.001
Part time	8 (9)	177 (8)	185 (8)		6 (8)	170 (9)	176 (9)	
Retired	32 (34)	951 (44)	983 (44)		23 (32)	849 (43)	872 (43)	
Unemployed	21 (22)	87 (4)	108 (5)		18 (25)	72 (4)	90 (4)	
Health insurance type, No. (%)								
Medicare	44 (45)	1013 (47)	1057 (47)	<.001	34 (47)	904 (46)	938 (46)	<.001
Private or HMO	37 (38)	1049 (49)	1086 (48)		29 (40)	993 (50)	1022 (50)	
VA or military	2 (2)	22 (1)	24 (1)		0 (0)	24 (1)	24 (1)	
Medicaid	4 (4)	23 (1)	27 (1)		4 (5)	20 (1)	24 (1)	
Other	4 (4)	23 (1)	27 (1)		5 (7)	13 (1)	18 (1)	
None	6 (6)	23 (1)	29 (1)		1 (1)	20 (1)	21 (1)	
Education, No. (%)								
Less than high school	23 (24)	168 (8)	191 (9)	<.001	20 (29)	144 (8)	164 (8)	<.001
High school graduate	25 (27)	385 (19)	410 (19)		20 (29)	349 (18)	369 (19)	
Some college	22 (23)	467 (23)	489 (23)		10 (14)	428 (22)	438 (22)	
College graduate	14 (15)	504 (24)	518 (24)		8 (11)	467 (25)	475 (24)	
Graduate or professional school	10 (11)	541 (26)	551 (26)		12 (17)	516 (27)	528 (27)	
Marital status, No. (%)								
Not married	32 (34)	379 (18)	411 (19)	<.001	24 (34)	352 (19)	376 (19)	<.001
Married	61 (66)	1681 (82)	1742 (81)		46 (66)	1549 (81)	1595 (81)	
TIBI, No. (%)								
0-2	18 (19)	618 (30)	636 (29)	<.001	11 (16)	595 (31)	606 (31)	.002
3-4	32 (34)	885 (43)	917 (42)		28 (40)	805 (42)	833 (42)	
≥5	44 (47)	570 (27)	614 (28)		31 (44)	511 (27)	542 (27)	
D’Amico risk group, No. (%)								
Low risk	31 (32)	979 (46)	1010 (45)	<.001	21 (29)	920 (47)	941 (46)	.01
Intermediate risk	37 (38)	839 (39)	876 (39)		34 (47)	755 (38)	789 (39)	
High risk	29 (30)	330 (15)	359 (16)		18 (25)	297 (15)	315 (15)	
PSA at diagnosis, corrected, No. (%)								
<4 (ng/ml)	15 (15)	415 (19)	430 (19)	<.001	11 (15)	386 (20)	397 (19)	.001
4-10 (ng/ml)	57 (59)	1487 (69)	1544 (69)		44 (60)	1376 (70)	1420 (69)	
10-20 (ng/ml)	16 (16)	202 (9)	218 (10)		12 (16)	168 (8)	180 (9)	
20-50 (ng/ml)	9 (9)	51 (2)	60 (3)		6 (8)	48 (2)	54 (3)	
Clinical tumor stage, No. (%)								
T1	71 (73)	1631 (76)	1702 (76)	.53	55 (75)	1497 (76)	1552 (76)	.90
T2	26 (27)	516 (24)	542 (24)		18 (25)	474 (24)	492 (24)	
Biopsy Gleason score, No. (%)								
6 or less	38 (39)	1112 (52)	1150 (51)	.008	26 (36)	1045 (53)	1071 (52)	.02
3 + 4	31 (32)	610 (28)	641 (29)		26 (36)	553 (28)	579 (28)	
4 + 3	9 (9)	212 (10)	221 (10)		12 (16)	186 (9)	198 (10)	
8-10	19 (20)	211 (10)	230 (10)		9 (12)	186 (9)	195 (10)	
Any ADT in year 1, No. (%)								
No	71 (75)	1857 (88)	1928 (87)	<.001	57 (79)	1712 (88)	1769 (88)	.03
Yes	24 (25)	263 (12)	287 (13)		15 (21)	237 (12)	252 (12)	
Treatment groups, No. (%)								
Surgery	43 (44)	1156 (54)	1199 (53)	.03	44 (60)	1074 (54)	1118 (55)	.24
Radiation	44 (45)	704 (33)	748 (33)		24 (33)	636 (32)	660 (32)	
Active surveillance	10 (10)	295 (14)	305 (14)		5 (7)	268 (14)	273 (13)	
EPIC-26 sexual function domain score at baseline, median (IQR)	60 (15, 89)	73 (38, 90)	73 (37, 90)	.04	60 (13, 85)	75 (38, 90)	73 (37, 90)	.06
EPIC-26 urinary incontinence domain score at baseline, median (IQR)	100 (73, 100)	100 (85, 100)	100 (81, 100)	.02	100 (67, 100)	100 (85, 100)	100 (81, 100)	.03
EPIC-26 urinary irritative domain score at baseline, median (IQR)	81 (62, 97)	88 (75, 100)	88 (75, 100)	.01	81 (58, 94)	88 (75, 100)	88 (75, 100)	.006
EPIC-26 bowel function domain score at baseline, median (IQR)	96 (75, 100)	100 (96, 100)	100 (92, 100)	<.001	92 (80, 100)	100 (96, 100)	100 (92, 100)	<.001
EPIC-26 hormonal domain score at baseline, median (IQR)	85 (70, 95)	95 (85, 100)	95 (85, 100)	<.001	75 (65, 92)	95 (85, 100)	95 (85, 100)	<.001
SF36 physical functioning at baseline, median (IQR)	80 (50, 100)	95 (85, 100)	95 (85, 100)	<.001	75 (50, 95)	95 (85, 100)	95 (85, 100)	<.001
SF36 emotional well-being at baseline, median (IQR)	68 (52, 88)	84 (72, 92)	84 (72, 92)	<.001	64 (52, 84)	84 (72, 92)	84 (72, 92)	<.001
SF36 energy and fatigue at baseline, median (IQR)	60 (40, 76)	75 (60, 85)	75 (60, 85)	<.001	60 (45, 75)	75 (60, 85)	75 (60, 85)	<.001
Perception of treatment effectiveness compared with expectations at 3 years, No. (%)								
A lot worse	7 (8)	37 (2)	44 (2)	<.001	—	—	—	
Better/same/a little worse	80 (92)	1964 (98)	2044 (98)		—	—	—	
Perception of treatment side effects compared with expectations at 3 years, No. (%)								
A lot worse	18 (21)	193 (10)	211 (10)	<.001	—	—	—	
Better/same/a little worse	68 (79)	1787 (90)	1855 (90)		—	—	—	
Perception of treatment effectiveness compared with expectations at 5 years, No. (%)								
A lot worse	—	—	—		5 (7)	24 (1)	29 (2)	<.001
Better/same/a little worse	—	—	—		64 (93)	1721 (99)	1785 (98)	
Perception of treatment side effects compared with expectations at 5 years, No. (%)								
A lot worse	—	—	—		13 (19)	175 (10)	188 (10)	.02
Better/same/a little worse	—	—	—		54 (81)	1554 (90)	1608 (90)	

^a^ Race and ethnicities included in the “other” category include American Indian, Native Alaskan, and patient responses of “other.” ADT = androgen deprivation therapy; EPIC-26 = Expanded Prostate Index Composite questionnaire; HMO = health maintenance organization; PSA = prostate-specific antigen; SF36 = 36-item Short Form Survey; TIBI = total illness burden index; VA = Veterans Affairs.

Prevalence of treatment regret was approximately 13% at 3- and 5-year follow-up (Table 2). Men who described large or very large financial burden were more likely to express some level of treatment regret (score >0 on treatment regret scale) on univariate analysis at that same time point. Clinically significant treatment regret (defined as a score >40) was present in 6% and 5% of patients experiencing large or very large overall financial burden at 3- and 5-year follow-up, respectively. Similar trends in treatment regret were observed in patients experiencing financial burden from treatment costs (4% at 3 years and 5% at 5 years), other health-care costs (5% at 3 years and 6% at 5 years), and health insurance costs (5% at 3 and 5 years).

**Table 2:** Association between financial toxicity and treatment regret at 3- and 5-year follow-up

Survey response	Treatment regret scale			Combined	*P*
	40-100	<40	0		
3-year follow-up survey					
Total No.	282	682	1276	2240	
Finances in general, No. (%)					
Very large or large burden	32 (11)	30 (4)	35 (3)	97 (4)	<.001
Neutral/small/very small/no burden	248 (89)	645 (96)	1235 (97)	2128 (96)	
Direct costs, No. (%)					
Treatment costs					
Very large or large burden	15 (5)	27 (4)	33 (3)	75 (3)	.04
Neutral/small/very small/no burden	263 (95)	647 (96)	1233 (97)	2143 (97)	
Other health-care costs					
Very large or large burden	21 (8)	24 (4)	35 (3)	80 (4)	<.001
Neutral/small/very small/no burden	259 (92)	653 (96)	1236 (97)	2148 (96)	
Health insurance costs					
Very large or large burden	23 (8)	20 (3)	39 (3)	82 (4)	<.001
Neutral/small/very small/no burden	257 (92)	654 (97)	1230 (97)	2141 (96)	
5-year follow-up survey					
Total No.	277	601	1184	2062	
Finances in general, No. (%)					
Very large or large burden	18 (7)	25 (4)	29 (2)	72 (4)	.002
Neutral/small/very small/no burden	252 (93)	568 (96)	1141 (98)	1961 (96)	
Direct costs, No. (%)					
Treatment costs					
Very large or large burden	13 (5)	29 (5)	23 (2)	65 (3)	.001
Neutral/small/very small/no burden	258 (95)	567 (95)	1147 (98)	1972 (97)	
Other health-care costs					
Very large or large burden	20 (7)	29(5)	19 (2)	68 (3)	<.001
Neutral/small/very small/no burden	253 (93)	566 (95)	1156 (98)	1975 (97)	
Health insurance costs					
Very large or large burden	17 (6)	30 (5)	18 (2)	65 (3)	<.001
Neutral/small/very small/no burden	255 (94)	566 (95)	1154 (8)	1975 (97)	

**Figure 2.**
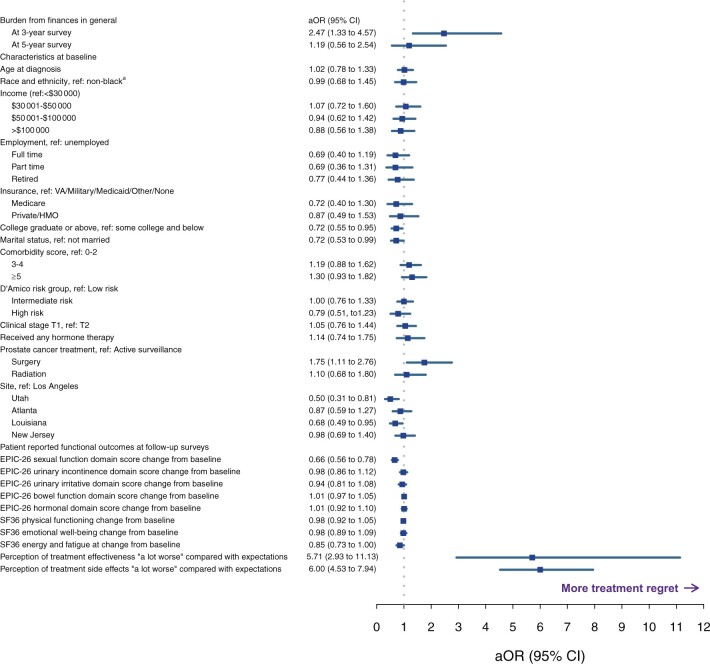
Multivariable longitudinal logistic regression with forest plot of the association between financial burden from finances in general and treatment regret after adjusting for potential confounders (**points** and **error bars** represent adjusted odds ratios and 95% confidence intervals, respectively). ^a^Non-Black race and ethnicities include Asian or Oriental or Pacific islander, American Indian or Native Alaskan, Latino or Hispanic or Mexican American, White or Caucasian, and patient-reported responses of “other.” aOR = adjusted odds ratio; CI = confidence interval; EPIC-26 = 26-item Expanded Prostate Index Composite questionnaire; HMO = health maintenance organization; SF36 = 36-item Short Form Survey; VA = Veterans Affairs.

In adjusted models accounting for relevant clinicopathologic, sociodemographic, and treatment characteristics as well as functional outcomes, overall financial burden was statistically significantly associated with treatment regret at 3 years (aOR = 2.47, 95% CI = 1.33 to 4.57; *P* < .01; Figure 2). This association was no longer statistically significant at 5-year follow-up (aOR = 1.19, 95% CI = 0.56 to 2.54; *P* = .66). Other variables associated with regret included treatment with surgery (compared with active surveillance; aOR = 1.75, 95% CI = 1.11 to 2.76), treatment efficacy worse than expectations (aOR = 5.71, 95% CI = 2.93 to 11.13), treatment side effects worse than expectations (aOR = 6.00, 95% CI = 4.53 to 7.94), change in EPIC-26 sexual function scores from baseline (more improvement vs less improvement; aOR = 1.52, 95% CI = 1.28 to 1.79), unmarried relationship status (aOR = 1.39, 95% CI = 1.01 to 1.89), and less than college education (aOR = 1.39, 95% CI = 1.05 to 1.82).

None of the remaining financial burden assessments, all of which assess direct costs (cost of treatment, other costs of health care, or cost of health insurance), were statistically significantly associated with treatment regret after adjusting for confounders (Supplementary Tables 2-4, available online). Of the direct cost measures, financial burden from treatment costs had the weakest relationship with treatment regret (OR = 1.02, 95% CI = 0.47 to 2.21). Regardless of which financial burden measure was included in the model, associations between treatment regret and surgical treatment, patient expectations of treatment efficacy and side effects, sexual function change from baseline, and education status remained statistically significant.

Of the baseline socioeconomic characteristics assessed, educational status was the only factor associated with treatment regret. Despite the observed relationship between regret and overall financial burden, models including either direct or indirect costs measurements revealed no association between treatment regret and income, insurance status, or employment status at any time point.

## Discussion

In this population-based sample of men undergoing management of localized prostate cancer, we found a statistically significant relationship between a patient’s overall financial burden and prostate cancer treatment regret at 3 years after adjusting for relevant clinicopathologic factors, treatment characteristics, and functional outcomes. Notably, direct patient costs such as those related to treatment, health insurance, and other costs of prostate cancer health care did not independently influence treatment regret. Financial burden at 5-year follow-up was rare and not associated with treatment regret. These findings address an important knowledge gap regarding the relationship between financial toxicity and treatment regret among cancer survivors.

Appropriate interpretation of these findings requires further discussion of the financial burden assessment employed in this study. Our 4-item questionnaire consisted of 3 questions that assessed direct costs related to treatment, health care, and health insurance. The remaining question assessed overall financial burden (finances in general), which includes both direct and indirect costs. Only overall financial burden was associated with treatment regret at 3-year follow-up, and this association was no longer statistically significant at 5-year follow-up. This may be a signal that the indirect costs of treatment are more impactful in these patients. Additionally, the attenuation of financial burden’s influence on regret at 5 years may be the result of decreasing indirect costs as treatment intensity lessens over time. For example, treatment and clinic visits, time off work, and decreased productivity are likely more prevalent early in prostate cancer management when patients are receiving active treatment and recovering from those treatments.

The association between financial burden and treatment regret observed in this study supports the inclusion of financial toxicity in shared decision making for localized prostate cancer. Treatment regret in men with localized prostate cancer ranges from 12% to 30% and has remained relatively stable over the past 30 years (15,18-21). Various deleterious effects of treatment regret on mental health and health-related quality of life have been described (1,2,20,22-24). Shared decision making aims to limit patient dissatisfaction through effective discussions of cancer-specific and functional outcome expectations. Numerous decision aids designed to enhance this process have been evaluated; however, none of these tools include descriptions of the financial toxicity associated with current treatment options. Similarly, financial implications of treatment are rarely included in patient-physician discussions (5).

Omission of financial toxicity in shared decision making may be, in part, because of the paucity of data assessing its prevalence and impact on prostate cancer patients. We previously reported relatively low rates (15% at enrollment) of financial burden because of treatment costs in this population that decreased over time (3% at 5-year follow-up) (7). These findings are consistent with assessments of out-of-pocket costs and objective measures of financial toxicity among commercially insured prostate cancer patients and those managed at Veterans Health Administration facilities (25-27). Less understood is the type and prevalence of indirect costs related to prostate cancer treatment, such as loss of time while traveling to or recovering from treatment, workplace absenteeism, reduced work productivity, increased burden on informal care givers, childcare expenses, and loss of income because of early retirement. Our study highlights the need for continued evaluation of such indirect costs in this population.

Interpretation of our findings is limited by the lack of granularity in our financial burden assessment. Specific indirect costs contributing to financial burden were unable to be identified based on the 4-question assessment. Nevertheless, this instrument was thoughtfully designed to capture many important facets of financial burden and has been used in other study populations. It should be noted that response rates differed at 3- and 5-year follow-up resulting in potential heterogeneity between respondents at each time point thereby limiting any conclusions regarding the evolution of the relationship between financial burden and regret over time. Still, 3-year survey response rates were high (95%) thereby limiting the magnitude of heterogeneity between respondents at subsequent survey time points. Additional limitations include lack of disease recurrence data that may confound our results, a relatively small number of patients experiencing both the exposure and outcome of interest, potential recall bias, and nonrespondent bias. However, a robust response rate of 72% was obtained, and we present the largest and only assessment of financial toxicity and prostate cancer treatment regret in the literature.

Additional strengths of our study include the assessment of important potential confounders that have been linked to treatment regret, such as socioeconomic factors, functional outcomes, and patient expectations, using validated instruments (1,18-24,28,29). Sexual function and accurate expectations of treatment efficacy and side effects remain important influencers of treatment regret, however, financial toxicity may further exacerbate patient dissatisfaction, especially early on in treatment recovery. Interestingly, studies have suggested that financial stress and worse symptom burden may be interrelated, although only associations between hormonal function and financial burden were observed in our study (30).

In conclusion, moderate levels of financial toxicity have been described in patients with localized prostate cancer, and out-of-pocket costs appear to be comparable between active treatment modalities. Still, for a subset of patients, finances loom large and can impact their treatment and recovery experiences. We observed a strong association between overall financial burden and treatment regret at 3-year follow-up that attenuated by 5 years. Additionally, financial burden from direct costs was not associated with treatment regret at any time point. When considering inclusion of financial toxicity in shared decision making, emphasis on intangible costs, especially early in management, may be warranted. Further research is needed to identify specific indirect costs that impact prostate cancer patients the most. Through these efforts, physicians may be able to better provide accurate expectations of treatment options to their patients and thereby limit treatment regret.

## Funding

This work was supported by National Cancer Institute of the National Institutes of Health (NIH/NCI: R01CA230352); Agency for Healthcare Research and Quality (1R01HS019356, 1R01HS022640 to DAB); and the Patient-Centered Outcomes Research Institute (CE-12-11-4667 to DAB). Data management was facilitated by Vanderbilt University’s Research Electronic Data Capture (REDCap) system, which is supported by the Vanderbilt Institute for Clinical and Translational Research grant (UL1TR000011 from NCATS/NIH).

## Notes

**Role of the funder:** The funder had no role in the design or conduct of the study, manuscript preparation, or decision to submit the manuscript for publication.

**Disclosures:** No conflicts of interest exist for any of the authors included on this manuscript.

**Authors contributions:** Conceptualization: DDJ, CJDW, DAB; Data curation: LCH, ZZ, TK; Formal Analysis: LCH, ZZ, TK; Funding acquisition: DAB, DFP; Investigation: KEH, MG, ASH, XCW, LEP, AS, MRC, MH, BBO, SHK, SG; Methodology: DDJ, CJDW, DAB, DFP; Project administration: KEH, DFP, DAB; Resources: KEH, MG, ASH, XCW, LEP, AS, MRC, MH, BBO, SHK, SG; Software: TK, ZZ; Supervision: DAB; Validation: LCH, ZZ, TK, DAB; Visualization: DDJ, LCH, ZZ, TK; Writing—original draft: DDJ; Writing—review & editing: CJDW, DAB, KEH, MG, ASH, XCW, LEP, AS, MRC, MH, BBO, SHK, SG, DFP.

**Acknowledgements:** We thank the men who participated in CEASAR and shared their experience. We thank the study managers, staff, and chart abstractors for their efforts in data collection and help with this study.

## Supplementary Material

pkac071_Supplementary_Data

## Data Availability

The data underlying this article cannot be shared because of the need to protect the privacy of individuals who participated in the study. All summary level data are included within the manuscript and Supplementary material.
