# Supplementary material for: The Association Between Financial Toxicity and Treatment Regret in Men With Localized Prostate Cancer
Source: JNCI Cancer Spectr. 2022 Oct 18;6(6):pkac071. doi: 10.1093/jncics/pkac071 (PMC9731205; doi:10.1093/jncics/pkac071)
Supplement: pkac071_Supplementary_Data [file pkac071_supplementary_data.pdf]

SUPPLEMENTARY MATERIAL:

Supplementary Table 1: Financial burden assessment instrument.

Overall, how much of a burden is your having prostate cancer on you and your family in each of the following areas?	
Our finances in general	-Very large burden<br>-Large burden<br>-Feel neutral<br>-Small burden<br>-Very small burden<br>-Not a burden
Our finances due to the cost of my treatment	-Very large burden<br>-Large burden<br>-Feel neutral<br>-Small burden<br>-Very small burden<br>-Not a burden
Our finances due to the other costs of health care for prostate cancer (such as visits to the doctor, etc.)	-Very large burden<br>-Large burden<br>-Feel neutral<br>-Small burden<br>-Very small burden<br>-Not a burden
Our finances due to the cost of my health insurance because I have prostate cancer	-Very large burden<br>-Large burden<br>-Feel neutral<br>-Small burden<br>-Very small burden<br>-Not a burden

Supplementary Table 2: Multivariable longitudinal logistic regression of the association between financial burden from "treatment costs" and treatment regret after adjusting for potential confounders.

	aOR (95% CI)	p value
**Burden from treatment costs**		
At 3-year survey	1.02 (0.47, 2.21)	0.96
At 5-year survey	0.78 (0.34, 1.82)	0.6
**Characteristics at baseline[1]**		
Age at diagnosis	0.99 (0.76, 1.30)	0.9
Race and ethnicity (ref: non-Black*)	0.99 (0.68, 1.44)	0.96
Income (ref: < $30,000)		
$30,001 - $50,000	1.00 (0.68, 1.490	0.99
$50,001 - $100,000	0.86 (0.57, 1.30)	0.5
> $100,000	0.80 (0.50, 1.26)	0.3
Employment (ref: unemployed)		
Full time	0.63 (0.37, 1.08)	0.09
Part time	0.63 (0.33, 1.20)	0.2
Retired	0.70 (0.40, 1.23)	0.2
Insurance (ref: VA/Military/Medicaid/Other/None)		
Medicare	0.70 (0.39, 1.26)	0.2
Private/HMO	0.83 (0.47, 1.47)	0.5
College graduate or above (ref: some college and below)	0.72 (0.55, 0.95)	0.02
Marital status (ref: not married)	0.71 (0.52, 0.98)	0.04
Comorbidity Score (ref: 0-2)		
3 - 4	1.23 (0.91, 1.66)	0.2
5 or more	1.35 (0.97, 1.90)	0.07
D'Amico risk group (ref: Low risk)		
Intermediate Risk	1.02 (0.77, 1.35)	0.9
High Risk	0.83 (0.53, 1.29)	0.4
Clinical stage T1 (ref: T2)	1.08 (0.79, 1.48)	0.7
Received any hormone therapy	1.17 (0.76, 1.78)	0.5
Prostate cancer treatment (ref: Active surveillance)		
Surgery	1.79 (1.13, 2.83)	0.01
Radiation	1.14 (0.70, 1.85)	0.6
Site (ref: LA)		
Utah	0.54 (0.33, 0.87)	0.01
Atlanta	0.90 (0.61, 1.32)	0.6
Louisiana	0.70 (0.50, 0.97)	0.03
NJ	1.04 (0.73, 1.48)	0.8

Patient reported functional outcomes at follow-up surveys[2]		
EPIC-26 sexual function domain score change from baseline	0.67 (0.57, 0.78)	<0.001
EPIC-26 urinary incontinence domain score change from baseline	0.98 (0.86, 1.12)	0.8
EPIC-26 urinary irritative domain score change from baseline	0.93 (0.80, 1.08)	0.3
EPIC-26 bowel function domain score change from baseline	1.01 (0.97, 1.05)	0.7
EPIC-26 hormonal domain score change from baseline	1.00 (0.91, 1.09)	0.97
SF36 physical functioning change from baseline	0.98 (0.92, 1.05)	0.6
SF36 emotional well-being change from baseline	0.99 (0.90, 1.10)	0.9
SF36 energy and fatigue at change from baseline	0.84 (0.71, 0.99)	0.04
Perception of treatment effectiveness "a lot worse" compared to expectations	5.92 (3.07, 11.42)	<0.001
Perception of treatment side effects "a lot worse" compared to expectations	6.00 (4.53, 7.95)	<0.001

[1]: Comparison is between the first quartile and the third quartile for continuous variables.

[2]: For all EPIC-26 domain score and SF36 score changes, the reported effects were for comparing the upper quartiles (more improving/less worsening) to the lower quartiles (less improving/more worsening) in domain score changes. For example, for the sexual domain, the comparison was between 0 (no change) and -42 (42-point worsening). For the SF36 emotional well-being, the comparison was between 8 (improving) and -4 (worsening).

*****non-Black race and ethnicities included: Asian/Oriental/Pacific islander, American Indian/Native Alaskan, Latino/Hispanic/Mexican-American, white/Caucasian, and patient-reported responses of "other."

Supplementary Table 3: Multivariable longitudinal logistic regression of the association between financial burden from "healthcare costs" and treatment regret after adjusting for potential confounders.

	aOR (95% CI)	p value
Burden from other healthcare costs		
At 3-year survey	1.85 (0.92, 3.72)	0.08
At 5-year survey	1.62 (0.77, 3.41)	0.2
Characteristics at baseline[1]		
Age at diagnosis	1.00 (0.77, 1.30)	0.99
Race and ethnicity (ref: non-Black*)	0.99 (0.67, 1.44)	0.94
Income (ref: < $30,000)		
$30,001 - $50,000	1.06 (0.71, 1.59)	0.8
$50,001 - $100,000	0.93 (0.61, 1.40)	0.7
> $100,000	0.87 (0.55, 1.38)	0.6
Employment (ref: unemployed)		
Full time	0.65 (0.38, 1.11)	0.1
Part time	0.65 (0.34, 1.24)	0.2
Retired	0.73 (0.42, 1.28)	0.3
Insurance (ref: VA/Military/Medicaid/Other/None)		
Medicare	0.69 (0.38, 1,24)	0.2
Private/HMO	0.82 (0.47, 1.44)	0.5
College graduate or above (ref: some college and below)	0.72 (0.54, 0.95)	0.02
Marital status (ref: not married)	0.72 (0.52, 0.98)	0.04
Comorbidity Score (ref: 0-2)		
3 - 4	1.20 (0.88, 1.62)	0.2
5 or more	1.30 (0.93, 1.81)	0.1
D'Amico risk group (ref: Low risk)		
Intermediate Risk	1.01 (0.76, 1.34)	0.95
High Risk	0.81 (0.52, 1,26)	0.4
Clinical stage T1 (ref: T2)	1.07 (0.78, 1.47)	0.7
Received any hormone therapy	1.15 (0.75, 1.76)	0.5
Prostate cancer treatment (ref: Active surveillance)		
Surgery	1.75 (1.10, 2.76)	0.02
Radiation	1.10 (0.68, 1.80)	0.7
Site (ref: LA)		
Utah	0.53 (0.33, 0.85)	0.01
Atlanta	0.88 (0.60. 1.29)	0.5
Louisiana	0.69 (0.50, 0.96)	0.03
NJ	1.00 (0.71, 1.43)	0.98

Patient reported functional outcomes at follow-up surveys[2]		
EPIC-26 sexual function domain score change from baseline	0.67 (0.57, 0.79)	<0.001
EPIC-26 urinary incontinence domain score change from baseline	0.98 (0.86, 1.11)	0.7
EPIC-26 urinary irritative domain score change from baseline	0.94 (0.81, 1.09)	0.4
EPIC-26 bowel function domain score change from baseline	1.01 (0.97, 1.05)	0.7
EPIC-26 hormonal domain score change from baseline	1.00 (0.92, 1.10)	0.9
SF36 physical functioning change from baseline	0.98 (0.92, 1.04)	0.5
SF36 emotional well-being change from baseline	0.99 (0.90, 1.09)	0.8
SF36 energy and fatigue at change from baseline	0.85 (0.72, 1.00)	0.05
Perception of treatment effectiveness "a lot worse" compared to expectations	5.79 (2.98, 11.21)	<0.001
Perception of treatment side effects "a lot worse" compared to expectations	5.99 (4.53, 7.94)	<0.001

[1]: Comparison is between the first quartile and the third quartile for continuous variables.

[2]: For all EPIC-26 domain score and SF36 score changes, the reported effects were for comparing the upper quartiles (more improving/less worsening) to the lower quartiles (less improving/more worsening) in domain score changes. For example, for the sexual domain, the comparison was between 0 (no change) and -42 (42-point worsening). For the SF36 emotional well-being, the comparison was between 8 (improving) and -4 (worsening).

*****non-Black race and ethnicities included: Asian/Oriental/Pacific islander, American Indian/Native Alaskan, Latino/Hispanic/Mexican-American, white/Caucasian, and patient-reported responses of "other."

Supplementary Table 4: Multivariable longitudinal logistic regression of the association between financial burden from "health insurance costs" and treatment regret after adjusting for potential confounders.

	aOR (95% CI)	p value
Burden from health insurance		
At 3-year survey	1.71 (0.89, 3.30)	0.1
At 5-year survey	1.34 (0.63, 2.86)	0.5
Characteristics at baseline[1]		
Age at diagnosis	0.99 (0.76, 1.29)	0.9
Race and ethnicity (ref: non-Black*)	1.00 (0.68, 1.45)	0.98
Income (ref: < $30,000)		
$30,001 - $50,000	1.07 (0.72, 1.59)	0.7
$50,001 - $100,000	0.93 (0.61, 1.40)	0.7
> $100,000	0.87 (0.55, 1.37)	0.5
Employment (ref: unemployed)		
Full time	0.64 (0.37, 1.08)	0.1
Part time	0.64 (0.33, 1.21)	0.2
Retired	0.71 (0.41, 1.25)	0.2
Insurance (ref: VA/Military/Medicaid/Other/None)		
Medicare	0.69 (0.39, 1.24)	0.2
Private/HMO	0.81 (0.46, 1.43)	0.5
College graduate or above (ref: some college and below)	0.72 (0.54, 0.95)	0.02
Marital status (ref: not married)	0.73 (0.53, 1.00)	0.05
Comorbidity Score (ref: 0-2)		
4-Mar	1.20 (0.89, 1.62)	0.2
5 or more	1.31 (0.94, 1.84)	0.1
D'Amico risk group (ref: Low risk)		
Intermediate Risk	1.01 (0.76, 1.33)	0.96
High Risk	0.80 (0.52, 1.25)	0.3
Clinical stage T1 (ref: T2)	1.05 (0.77, 1.45)	0.8
Received any hormone therapy	1.13 (0.73, 1.74)	0.6
Prostate cancer treatment (ref: Active surveillance)		
Surgery	1.73 (1.10, 2.74)	0.02
Radiation	1.10 (0.68, 1.79)	0.7
Site (ref: LA)		
Utah	0.51 (0.31, 0.82)	0.01
Atlanta	0.87 (0.60, 1.28)	0.5
Louisiana	0.69 (0.49, 0.96)	0.03
NJ	0.99 (0.70, 1.42)	0.97

Patient reported functional outcomes at follow-up surveys[2]		
EPIC-26 sexual function domain score change from baseline	0.67 (0.57, 0.78)	<0.001
EPIC-26 urinary incontinence domain score change from baseline	0.98 (0.86, 1.11)	0.7
EPIC-26 urinary irritative domain score change from baseline	0.94 (0.81, 1.09)	0.4
EPIC-26 bowel function domain score change from baseline	1.01 (0.97, 1.05)	0.7
EPIC-26 hormonal domain score change from baseline	1.00 (0.91, 1.09)	0.99
SF36 physical functioning change from baseline	0.98 (0.91, 1.04)	0.5
SF36 emotional well-being change from baseline	0.99 (0.89, 1.09)	0.8
SF36 energy and fatigue at change from baseline	0.85 (0.72, 1.00)	0.05
Perception of treatment effectiveness "a lot worse" compared to expectations	5.77 (2.97, 11.22)	<0.001
Perception of treatment side effects "a lot worse" compared to expectations	6.00 (4.53, 7.95)	<0.001

[1]: Comparison is between the first quartile and the third quartile for continuous variables.

[2]: For all EPIC-26 domain score and SF36 score changes, the reported effects were for comparing the upper quartiles (more improving/less worsening) to the lower quartiles (less improving/more worsening) in domain score changes. For example, for the sexual domain, the comparison was between 0 (no change) and -42 (42-point worsening). For the SF36 emotional well-being, the comparison was between 8 (improving) and -4 (worsening).

*****non-Black race and ethnicities included: Asian/Oriental/Pacific islander, American Indian/Native Alaskan, Latino/Hispanic/Mexican-American, white/Caucasian, and patient-reported responses of "other."

Supplementary Figure 1: Survey administration schedule for assessment of functional outcomes (EPIC-26), financial burden (developed by study psychometrician), and treatment regret (instrument developed by Clark et al and validated in prostate cancer patients).

Survey	Baseline	6 months	1 year	3 years	5 years
EPIC-26	x	x	x	x	x
Financial Burden	x	x	x	x	x
Treatment Regret				x	x
